# Learning from Mother Nature: Innovative Tools to Boost Endogenous Repair of Critical or Difficult-to-Heal Large Tissue Defects

**DOI:** 10.3389/fbioe.2017.00028

**Published:** 2017-04-28

**Authors:** Ranieri Cancedda, Sveva Bollini, Fiorella Descalzi, Maddalena Mastrogiacomo, Roberta Tasso

**Affiliations:** ^1^Biorigen Srl, Genova, Italy; ^2^Department of Experimental Medicine, University of Genova, Genova, Italy; ^3^IRCCS AOU San Martino-IST National Institute of Cancer Research, Genova, Italy

**Keywords:** tissue regeneration, tissue healing, platelet factors, innate immunity, angiogenesis, stem cells, cell reprogramming

## Abstract

For repair of chronic or difficult-to-heal tissue lesions and defects, major constraints exist to a broad application of cell therapy and tissue engineering approaches, i.e., transplantation of “*ex vivo*” expanded autologous stem/progenitor cells, alone or associated with carrier biomaterials. To enable a large number of patients to benefit, new strategies should be considered. One of the main goals of contemporary regenerative medicine is to develop new regenerative therapies, inspired from Mother Nature. In all injured tissues, when platelets are activated by tissue contact, their released factors promote innate immune cell migration to the wound site. Platelet-derived factors and factors secreted by migrating immune cells create an inflammatory microenvironment, in turn, causing the activation of angiogenesis and vasculogenesis processes. Eventually, repair or regeneration of the injured tissue occurs *via* paracrine signals activating, mobilizing or recruiting to the wound site cells with healing potential, such as stem cells, progenitors, or undifferentiated cells derived from the reprogramming of tissue differentiated cells. This review, largely based on our studies, discusses the identification of new tools, inspired by cellular and molecular mechanisms overseeing physiological tissue healing, that could reactivate dormant endogenous regeneration mechanisms lost during evolution and ontogenesis.

## Introduction

Over the last two decades, significant progresses have been made by transplantation of “*ex vivo*” expanded autologous stem/progenitor cells, mostly associated with biomaterials, for the repair of chronic or difficult-to-heal tissue lesions and defects caused by disease, trauma, or tumor surgery. Our lab was among the pioneers in applying a tissue engineering approach for repairing and reconstructing human epithelia in burned patients (De Luca et al., 1989), as well as urethra for congenital malformations (Romagnoli et al., 1990), corneal epithelium in eye chemical burns (Pellegrini et al., 1997), and long-bone defects in trauma outcome (Quarto et al., 2001). However, despite these successes, we must acknowledge that major constraints exist for the broad application of this approach, including complexity and morbidity of surgical procedures, and logistics and high costs of the cell isolation and expansion in highly qualified structures. Therefore, it appears that “classical” tissue engineering should be considered only in extreme and very critical situations. Hence, in order to enable a large number of patients to benefit, new strategies should be developed. In the last decades, thanks to the recent progresses in chemistry, biochemistry, and pharmacology, we have seen the development and application of a large number of drugs and devices aimed at the treatment of symptoms, blocking unwanted pathways and, in the case of infectious diseases, fighting the responsible microorganisms. Nonetheless, we are currently facing a dramatic change in the therapeutic approach to pathologies and diseases. Indeed, the present and the next years challenge is to fully restore the physiological status of a diseased organism and to completely regenerate tissue and organs when so seriously affected that treatments cannot be limited to the repression of symptoms. Unfortunately, the body self-regeneration capacity has been lost during evolution. While a fragment of planaria can generate a whole new individual, newts can regrow a limb, lizards their tail, crabs can develop a new claw, and zebrafishes can rekindle the heart, we, as adult humans, cannot rely on a full regeneration of a lost part of the body since restorative potential is limited to the human fetus or to a very early postnatal stage. Thankfully, evolution granted us with a much better brain than a planaria, a newt, a lizard, a crab, and a zebrafish. Thus, we must use our brain to develop products and procedures able to reactivate the regeneration mechanisms lost during evolution.

The common core concept discussed in this article, largely based on studies of our laboratory, focuses on the identification of tools to reactivate or stimulate dormant endogenous regeneration mechanisms, since this probably represents the main goal of contemporary regenerative medicine.

## Endogenous Regeneration

### Endogenous Regeneration Potential Is Lost during Phylogeny and Ontogeny

Regeneration is the structural and functional restoration of a missing part of the body. Regeneration can occur at different levels of biological organization. The newt is able to regenerate the limbs throughout its lifespan. When a limb is amputated, a cell mass called *blastema* is formed at the stump from which a new functional limb will be shaped. The newt switches the cellular mechanism for limb regeneration from a stem/progenitor-based mechanism (larval mode) to a dedifferentiation-based one (adult mode) (Tanaka et al., 2016). At the larval stage, new tissues of the regenerated limb originate from stem/progenitor cells recruited to the *blastema*. Conversely, after metamorphosis, differentiated cells of the limb tissues revert to a pre-differentiation stage and contribute to the *blastema* formation, eventually giving rise to the fully regenerated limb. Cells from the different adult limb tissues, such as skin, bone, muscle, and nerves strictly regenerate the same kind of tissues components. Most of the other amphibian models, analyzed so far, regenerate limbs at the larval stage, but this capacity is lost after metamorphosis. However, stem/progenitor cells are still present in the tissues, such as satellite cells within the skeletal muscles. This suggests that the potential of stem/progenitor cells to be mobilized into the *blastema* is lost during metamorphosis. Indeed, in *Xenopus laevis*, it has been reported that the expression of hepatocyte growth factor, which can recruit satellite cells into the *blastema*, decreases during metamorphosis (Alvarado and Tsonis, 2006; Beck et al., 2009). An increasing number of experimental evidences suggest that regeneration recapitulates the original structure development (Nacu and Tanaka, 2011). For example, it has been reported that during limb regeneration several genes originally expressed during limb development are reactivated. Hence, the contemporary working paradigm in regenerative medicine is based on the idea that tissue regeneration in the adult can be strengthened by the reactivation of those same embryonic mechanisms that contributed to tissue morphogenesis in first instance.

Although mammals are unable of regenerating limbs and tails, a regenerative potential has been described by different authors in several tissues and organs of mammalian embryos and fetuses (Colwell et al., 2005; Nakada et al., 2015). Fetuses can regrow almost anything that gets damaged while in the womb. Instead, liver regeneration and the regrowth of the amputated digit tips, this last occurring in children in the first decade and before sexual maturity, are unique examples of after-birth regeneration in humans (Kisch et al., 2015). The mouse digit tip model imitates very well the endogenous regenerative response of the human digit tip (Simkin et al., 2013), and it was used to show the role played by growth factors, such as BMP2 and BMP7, present in the wound exudate. Interestingly, Seifert et al. (2012) reported the first example of a large portion of skin regeneration in mammals. In the African spiny mice, after a skin autotomy to elude capture by predators, complete skin regeneration occurs in record time. This may indicate that mammal regeneration may not be a distant hope as we had feared.

However, although cellular and molecular mechanisms of regeneration are currently being investigated in a variety of contexts (Tanaka and Reddien, 2011), our understanding of the epigenetic reprogramming controlling regeneration in the different species is only at the beginning (Katsuyama and Paro, 2011). In humans and mice, an efficient endogenous regenerative response fails to occur after a substantial injury, such as a myocardial infarction. On the contrary to mammals, in zebrafish, an amputated, cryoinjured, or infarct heart can fully regenerate by reactivation of cardiac progenitor cells and the dedifferentiation, proliferation, and maturation of residing cardiomyocytes (Kikuchi et al., 2010). Aguirre et al. (2014) identified miR-99/100 and Let-7a/c and their protein targets SMARCA5 and FNTB as critical regulators of cardiomyocyte dedifferentiation and heart regeneration in zebrafish. Despite the inability to efficiently activate endogenous regenerative mechanisms, the molecular regulators of this response are conserved in mammals. The manipulation of this molecular machinery in mice resulted in cardiomyocyte dedifferentiation and improved heart function after injury (Aguirre et al., 2014). Moreover, it was recently shown that the rodent heart still maintain full myocardial regenerative capacity, but strictly limited to the first week of life (Porrello et al., 2011), possibly suggesting that such molecular machinery might remain quiescent and dormant due to epigenetic differences among different species and/or during aging.

### Lesson Learned from Regeneration in Different Animal Species

Basically, all organisms have the ability to regenerate something. This process occurs to a greater extent in lower organisms, such as flatworms, and in invertebrates, like starfishes and earthworms. Regeneration is much more limited in higher organisms. Although it is quite obvious that the regeneration potential is lost during evolution, the mechanisms by which the natural selection operated has not been always clear since many incongruities exist among close species. As an example, only earthworms, and not the close species leech, can replace lost parts of the body. Some insects can regrow lost legs, but others insect cannot. Bony fishes, but not cartilaginous fishes, can regenerate fins. Among amphibians, salamanders and newts regenerate their legs, but frogs and toads do not [reviewed in Simon and Tanaka (2013)].

However, in order for regeneration to occur, there must be a source of *blastema* (undifferentiated cells) and regeneration must be activated by an external physiological stimulus. Undifferentiated cells are more abundant in the embryonic and fetal life that in adults and this could explain the greater regeneration potential of embryos and fetuses compared to adults. Alternatively, in adults, undifferentiated cells could derive from the remnants of the original structure (Tanaka et al., 2016). The physiological stimulus could be mediated by a nerve supply (Peadon and Singer, 1965; Simões et al., 2014), could be molecules such as hormones and growth factors [reviewed in Nacu and Tanaka (2011)], or could depend upon an interaction between cells of different tissues, including epidermal/mesenchymal interactions. It has been suggested that a key role in these interactions between cells of different tissues is played by matrix metalloproteases (MMPs) (Santosh et al., 2011).

Moreover, the natural selection pressure in response to other needs of the animal and the way by which wound heals have to be considered. Differences observed in amphibians could be related to the fact that legs are not very useful for the movements of salamander and newt in water, whereas frogs and toads are dependent on their legs to move. Given their size and complex structure, higher vertebrates have the urgent need of a rapid wound closure and damage repair in case of injury, and this occurs through the formation of a thick scar tissue to the detriment of full regeneration. As an example, in a skin injury, the scar tissue could impair the contact between epidermis and underlying tissues which is required to trigger the rise of *blastema* like cells, key players in the regeneration process.

### Mechanisms and Phases of Physiological Wound Healing in Humans

**Figure 1:**
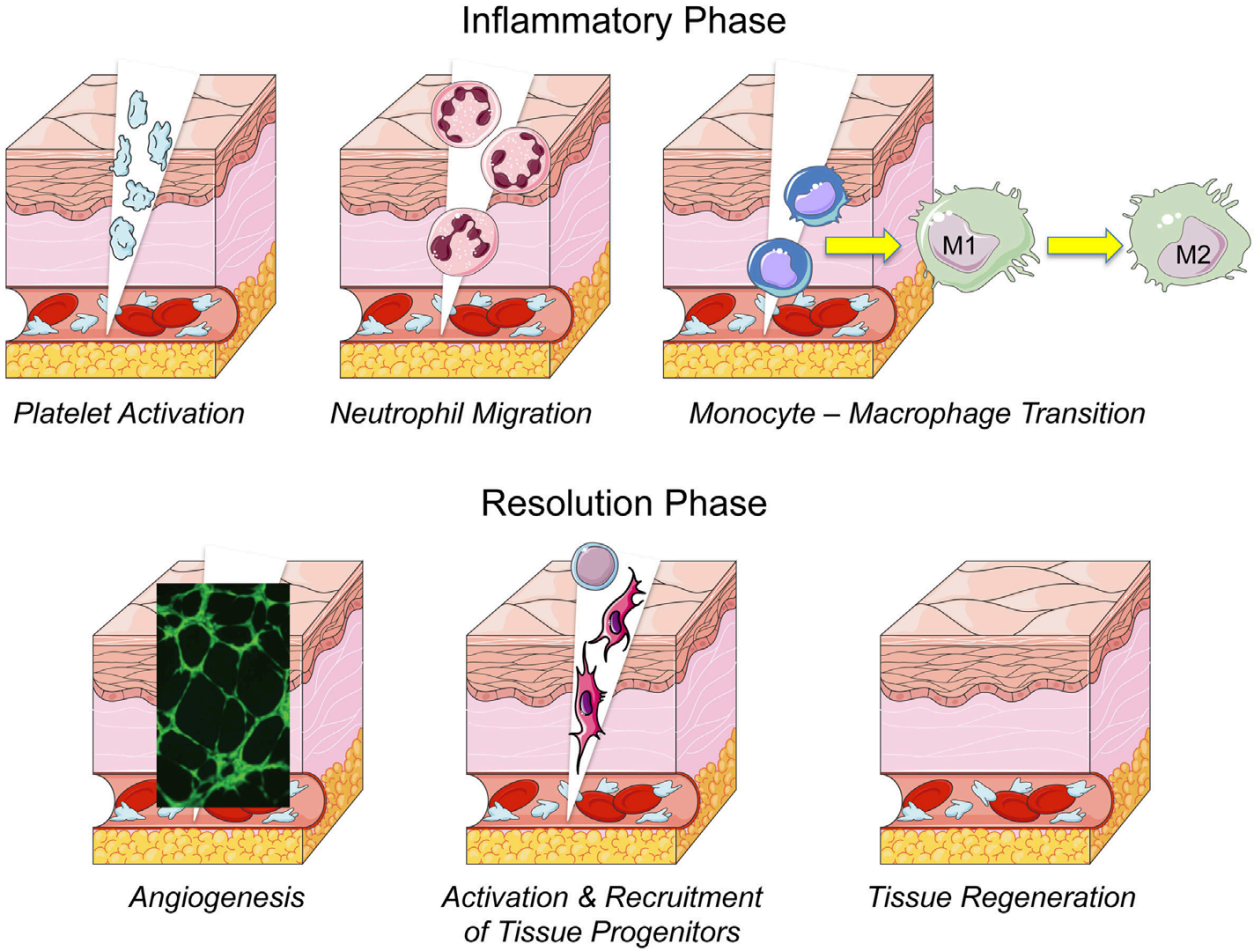
**Main events and phases of the wound healing process**.

To develop new regenerative therapies in humans, we should learn from Mother Nature and expand our knowledge about cellular and molecular mechanisms overseeing physiological tissue healing. Although every tissue has a specific response to healing, there is a common initial stage after the damage that is characterized by hematoma, clot formation, and platelet activation (Gurtner et al., 2008; Nurden et al., 2008; Widgerow, 2011). Main events occurring during a physiological healing are depicted in Figure 1. In all tissues—apart from hyaline cartilage—upon injury, platelets form a plug that stabilizes the clot within a fibrin meshwork (Foster et al., 2009). When platelets get activated by the contact with the injured tissue, or by thrombin and calcium, they release a variety of growth factors and bioactive molecules *via* a degranulation process. Platelet released factors and molecules play a significant role in triggering the healing process and are crucial for regulating immune cell migration (Morrell et al., 2014). Immune cells and their secreted factors are key master regulators for progression of the physiological response to injury (Shi et al., 2003). In fact, hematoma, immune cells and their secreted factors create an inflammatory microenvironment, at the site of the wound, attracting local and circulating endothelial, mesenchymal, and epithelial cells (Bussolino et al., 1989; Rabbany et al., 2003; Tasso et al., 2013; Ulivi et al., 2014). This results in angiogenesis and vasculogenesis activation, i.e., new vessel formation by sprouting of pre-existing vessels and from recruited endothelial progenitors, respectively. Following this first inflammatory phase, tissue repair or regeneration may occur *via* paracrine signals mobilizing or recruiting, to the wound site, cells with healing potential, such as stem cells, progenitors, and immature cells derived from resident differentiated cell reprogramming.

### Targeting the Tissue Repair/Regeneration Process by Platelet Derivatives

Given the triggering role played by platelets during tissue healing, local application of autologous platelet-rich plasma (PRP) has been proposed as source of factors and nutrients to treat chronic or acute, often severe, damages in different organ districts. These include chronic skin ulcers (Martínez-Zapata et al., 2009), articular cartilage damage (Dold et al., 2014), bone and tendon lesions (Alsousou et al., 2009; Intini, 2009), peripheral nerve wound (Zheng et al., 2014), cardiac muscle injury (Vu et al., 2015), plastic and maxillofacial applications (Marx et al., 1998; Martínez-Zapata et al., 2009). PRP has also been used for the treatment of injuries in professional sportspersons (Taylor et al., 2011; Dold et al., 2014) and in racing animals (Waselau et al., 2008; Witte et al., 2016). The use of PRP or other platelet-derived components relies on the reactivation of latent endogenous regeneration mechanisms. *In vitro* experiments from our group demonstrated that, following an initial transient enhancement of the inflammatory response (NF-kB activation, COX-2 induction, and secretion of pro-inflammatory cytokines), PRP favored the establishment of an anti-inflammatory microenvironment *via* prostaglandin E2 (PGE_2_) production (Ruggiu et al., 2013). An upregulation of the proliferative and survival pathways ERKs and Akt and the cell cycle reactivation in quiescent cells *via* induction of Cyclin D1 and phosphorylation of retinoblastoma was also observed. A 24 h treatment was sufficient to trigger all processes. Moreover, PRP can promote recruitment of neutrophils and macrophages, which, in turn, due to their paracrine activity, not only enhance endothelial cell recruitment and improve vascularization, but also attract and stimulate proliferation of circulating and/or locally resident mesenchymal and epithelial stem/progenitor cells, thus enhancing whole tissue repair (Pierce et al., 1989). Interesting, PRP has also been shown to exert an antinociceptive activity by a peripheral endocannabinoid-related mechanism (Descalzi et al., 2013).

Platelet-rich plasma has the advantage of being a concentrated cocktail of factors, present in ideal relative proportions, and necessary for healing. Among them are platelet-derived growth factors (PDGF-A and PDGF-B homo- and hetero-dimers), vascular endothelial growth factor (VEGF), epidermal growth factor (EGF), heparin binding EGF-like growth factor (HB-EGF), fibroblast growth factor (FGF), transforming growth factor beta (TGF-β), and interleukin-8 (IL-8). Notably, the expression of these factors is spatiotemporally regulated during wound healing. Indeed, the missed expression of some of these factors could delay the wound process (Martin, 1997; Werner and Grose, 2003; Gurtner et al., 2008). PDGF plays a key role both at an early and a late stage of healing. In the early phases, PDGF recruits to the wound bed neutrophils, monocytes/macrophages, and participates in the macrophage activation. At a later stage, it stimulates pericyte and fibroblast recruitment and proliferation and enhances matrix deposition (Martin, 1997; Werner and Grose, 2003). PDGF also induces myofibroblast differentiation and stimulates fibroblasts to contract collagen fibers (Werner and Grose, 2003; Martino et al., 2014, 2015). PDGF plays a key role also in angiogenesis and the combined delivery with VEGF can normalize defective angiogenesis (Martino et al., 2015). VEGF-A, which is secreted in high amount by keratinocytes and macrophages, is a master regulator of physiological and pathological angiogenesis and vasculogenesis acting on the granulation tissue formation (Martino et al., 2014, 2015). FGF-2, present at significant concentration in the initial wound fluid, stimulates wound repair by promoting fibroblast and endothelial cell proliferation, migration, and differentiation and in synergy with VEGF it also sustains angiogenesis (Werner and Grose, 2003; Gurtner et al., 2008). EGF induces the proliferation and cell migration of keratinocytes during the re-epithelialization process (Martin, 1997). HB-EGF is a keratinocyte and fibroblast mitogen and also promotes cell migration (Martin, 1997; Werner and Grose, 2003). TGF-β is a potent chemoattractant for neutrophils, macrophages, and fibroblasts and stimulates formation of granulation and re-epithelialization (Werner and Grose, 2003). IL-8 is involved in angiogenesis and re-epithelialization and inhibits wound contraction (Werner and Grose, 2003).

Platelet-rich plasma is usually prepared from the patient whole blood (autologous PRP) by separating the red blood cells and concentrating the platelets and other components of plasma. However, blood of different individuals can present a significant variability in platelet concentration (Weibrich et al., 2002). Moreover, obtaining autologous fresh PRP from the patient’s blood may be unwieldy. A standardized PRP-production procedure, starting from pools of human-certified buffy coats has been proposed to reduce PRP variability and to obtain a PRP with a well-defined platelet and growth factor concentration (Muraglia et al., 2014, 2015). Indeed, an allogenic source of platelets could offer further benefits including an “off the shelf,” quality controlled, and safe product. In addition, this could result in the use of buffy coats otherwise discarded after fractionation of humanitarianly donated blood. Given the triggering role of platelets in the tissue healing process, the use of their derived compounds was proposed also as cell culture medium supplements. Indeed, cell cultures were exploited to investigate molecular mechanisms and pathways activated in cells exposed to platelet derivatives (Ruggiu et al., 2013; Muraglia et al., 2014, 2015). Indeed, the effect of the platelet lysates (PLs) on cell proliferation and differentiation reprogramming was shown in different types of cells [also reviewed in Xie et al. (2014), Tobita et al. (2015), and Astori et al. (2016)].

### Guiding Innate Immune Response, Inflammation, and M1/M2 Macrophage Balance

Innate immune response molecules play an important role during regeneration of planaria, an organism that does not have an adaptive immune system (Peiris et al., 2014). Instead, an adaptive immune system is present in salamander; nonetheless, investigations on salamander scar-free repair and regeneration revealed the role of factors common to the planaria innate immune system (Godwin and Rosenthal, 2014). Thus, innate immune signaling may be crucial for the migration of proper cell types to the wound site. In humans, studies of healing in autoimmune patients indicated that a proper balance of immune response must be maintained during wound repair (Loots et al., 1998). Other studies suggested that a more developed immune system may be the cause of scaring and of a reduced regenerative capacity during wound repair (Peiris et al., 2014).

Neutrophils are the first inflammatory cells recruited to the wound site. They are chemoattracted by factors supplied by platelets, such as VEGF-A, and by the hypoxic microenvironment (de Oliveira et al., 2016). In the complex interplay represented by the wound healing niche, neutrophils are not only necessary to “clean” the injured area from bacteria and apoptotic cells but also to produce cytokines, growth factors, and other soluble mediators activating other cells present in the wound (Wilgus et al., 2013).

Monocytes, being the second wave of recruited cells, can be considered the most relevant immune cell population in the wound healing niche. Monocyte depletion impairs the repair/regenerative processes with a significant delay in wound healing parameters, including epithelialization and decreased neovascularization (van Amerongen et al., 2007; Brancato and Albina, 2011). Resident macrophages are a little fraction compared to macrophages derived from monocytes recruited to the wound site from the peripheral blood (PB). There are two main types of macrophages, namely, M1 and M2 (Murray et al., 2014). M1 macrophages (sometimes referred to as classically activated or pro-inflammatory macrophages) are characterized by iNOS expression, high level of IL-12, and low level of IL-10 secretion. M2 macrophages (also referred to as alternatively activated or anti-inflammatory macrophages) secrete high levels of IL-10 and TGF-β, low levels of IL-12, and express Arginase I, as well as multiple receptors, such as CD163, CD206, RAGE, and other scavenger receptors (Rath et al., 2014). M2 macrophages contribute to quench the inflammatory phase during healing *via* the release of anti-inflammatory molecules (Mantovani et al., 2013). A population of cardiac macrophages as mediators of heart repair and regeneration was identified after a myocardial infarction in 1-day-old neonatal mice (Aurora et al., 2014).

Recent findings indicate that macrophages are not just participants, but are coordinators of early wound healing events. Indeed, the sequential activation of macrophages to M1 and M2 phenotype orchestrate adult tissue regeneration and support the concept that inflammation, through macrophage activation, controls tissue precursor cell fate and coordinates tissue repair (Saclier et al., 2013; Das et al., 2015). In this context, we investigated the effect of the medium conditioned by mesenchymal stem cells (MSCs), previously exposed to a wound microenvironment, in promoting the macrophage polarization from an M1 to an M2 phenotype. Before the medium collection, MSCs were stimulated by molecules released by platelets and pro-inflammatory cytokines, the two main players at the onset of the wound healing process. This led to a progressively increasing concentration of PGE_2_ in the MSC medium, promoting the macrophage switch from a pro-inflammatory to an anti-inflammatory phenotype (Tasso et al., 2013; Ulivi et al., 2014). Moreover, other authors reported that a platelet supernatant suppressed the inflammatory responses of bone marrow-derived macrophages *via* negative regulation of NF-kB signaling, overall leading to lowered expression of iNOS and enhanced l-arginine catabolism by Arginase-1 (Ando et al., 2016). Additional *in vitro* experiments of our group showed that, during the initial enhancement of the inflammatory phase, PRP polarizes the monocyte differentiation also toward an anti-inflammatory subset of dendritic cells with some similarities to M2 macrophages thus giving additional evidence of PRP ability to modify the wound healing microenvironment (Papait et al., 2016).

### Supporting Neovascularization Phase during the Healing Process

The inflammatory phase is followed by a proliferation phase beginning with the formation of a granulation tissue. Granulation tissue, i.e., the combination of a new undifferentiated connective tissue and tiny blood vessels, forms on the wound surface and fills wounds of almost any size. New vessel formation, eventually leading to the restoration of the vascular network, can occur by (i) angiogenesis, i.e., the formation of new vessels through the sprouting of the pre-existing vessels, or by (ii) vasculogenesis, i.e., the formation of new vessels through the recruitment of endothelial progenitors circulating in the PB (Velazquez, 2007; Olfert et al., 2016). In both cases, the new vessels must be stabilized through the association of cells of mesenchymal origin, namely, pericytes, otherwise, they are rapidly resorbed within few days from their formation (Armulik et al., 2011).

The changes occurring within the wound site during the first days after injury promote both types of processes. The initial inflammatory microenvironment stimulates recruitment, adhesion, and homing of endothelial progenitors forming endothelial cell tubular structures first, and then of smooth muscle cells and pericytes wrapping around the endothelial cells, thus contributing to the newly formed capillary maturation and stabilization. In particular, platelets and innate immunity cell secretome are enriched of factors acting on endothelial cells of pre-existing vessels, hence activating endothelial cell proliferation and expression of proteases—mainly MMPs such as MT-MMP1—digesting the surrounding extracellular matrix to allow migration of the proliferating endothelial cells and formation of vessel sprouts connecting to other vessels. At this time, a critical microenvironment change is represented by the O_2_ supply reduction resulting in the activation of hypoxia-inducible factors, which in turn induce the expression of master angiogenesis regulating factors, including VEGF, and the FGF family, in particular FGF-1 and FGF-2 (Krock et al., 2011). Other factors playing a major role at this stage are Angiopoietins 1 and 2, Class 3 Semaphorins, members of the TGF-β superfamily, and the PDGF, which is also secreted by endothelial cells and promotes the migration of pericytes to the growing capillaries.

Sometimes angiogenesis can occur through a less understood mechanism named “splitting angiogenesis,” where a new vessel is formed by the splitting of a pre-existing blood vessel in two (Olfert et al., 2016). This allows an increase in the number of capillaries without necessarily a corresponding increase in the number of endothelial cells and could possibly be more energy-efficient than sprouting (Ji et al., 2006).

### Exploiting Stem and Progenitor Cell Secretome and Released Extracellular Vesicles for Regenerative Purposes

Until recently, stem cell therapy for tissue regeneration was based on the assumption that transplanted stem cells could directly contribute by transdifferentiation to the new tissue formation. However, an increasing body of experimental evidences suggests that the benefits obtained following stem cell implantation are mostly related to their paracrine activity, rather than a direct involvement in the tissue replacement. As result of this change in perspective, several studies are now specifically focusing on the stem cell secretome, as the whole of soluble paracrine factors, including growth factors, cytokines, and chemokines that the cells can release locally, thus contributing to the positive ending of the healing process.

In this scenario, our group was interested in characterizing the potential as regeneration inducer of the secretome fetal stem cells, such as the c-kit + human amniotic fluid stem cells (hAFS). Indeed, being fetal stem cells developmentally much younger and more immature compared to adult ones, and harboring a stronger proliferative potential, hAFS may represent an ideal source to exploit for future therapy (De Coppi et al., 2007). We showed that hAFS enhanced tissue repair by recruiting host progenitor cells at the site of *in vivo* transplantation (Mirebella et al., 2011). Moreover, hAFS secreted discrete paracrine angiogeneic factors, such as VEGF, IL-8, MCP-1, and SDF-1, triggering neoangiogenesis and sustaining tissue regeneration in a rat hind-limb ischemic model (Teodelinda et al., 2011). These encouraging results were further confirmed in a rat model of ischemic full-thickness skin flap elevated on the epigastric region, where the hAFS secretome induced both the activation of the local resident endothelial cells and the recruitment of endothelial progenitor cells (EPCs) (Mirabella et al., 2012). The powerful paracrine potential of AFS was also demonstrated by other independent studies, showing that fetal cells improved survival and clinical outcome in a preclinical rat model of necrotizing enterocolitis, decreased gut damage, and improved tissue structure and function mainly *via in situ* paracrine effects (Zani et al., 2014). Moreover, mouse GFP + AFS, injected in a chronic mouse model of skeletal muscle atrophy, enhanced muscle strength and improved the survival rate of the affected animals while restoring the muscle niche possibly *via* modulatory influence (Piccoli et al., 2012).

The adult mammalian heart has a very limited repair capacity when facing severe injury, congenital defects, or aging. This is due to a combination of tissue intrinsic causes, such as poor cardioprotection, defective repair, and extremely limited myocardial renewal potential. Benefits resulting from stem cell transplantation were observed by different authors, although controversial results were also reported (Toma et al., 2002; Murry et al., 2004; Wei et al., 2012). Indeed, the healing effects obtained following stem cell therapy were mainly explained as the result of the transplanted cell paracrine activity counteracting cardiovascular cell apoptosis and fibrosis, while modulating inflammation, improving neoangiogenesis, activating cardiac progenitor resident cells, and significantly improving cardiac function. In some initial studies, adult MSCs were transplanted (Gnecchi, 2006; Noiseux et al., 2006; Hatzistergos et al., 2010). However, a recent study showed that implanted hAFS exerted a remarkable cardioprotective influence on the ischemic heart of rats undergoing ischemia/reperfusion injury and, within 2 h, dramatically reduced the infarct size, suggesting a paracrine activity of the implanted cells. This hypothesis was confirmed by injecting the cell-conditioned medium (Bollini et al., 2011) and showing that the hAFS secretome was enriched with cardioactive factors, among which the small peptide thymosin beta 4, a well-known master regulator of epicardial progenitor cells activation (Smart et al., 2012) as well as a powerful cardioprotective agent (Bock-Marquette et al., 2004). More recently, our group characterized the cardioprotective mechanisms of the hAFS secretome also in a chemotherapy-derived model of cardiotoxicity. Indeed, the paracrine factors within the medium conditioned by hAFS prestimulated by a hypoxic environment exposure significantly counteracted doxorubicin-induced premature senescence and apoptosis of cardiomyocytes and cardiac progenitor cells, by inducing on the target cells an autocrine feedback based on production of pro-survival chemokines and cytokines consequent to the activation of the pAkt/PI3K pathway (Lazzarini et al., 2016).

Also transplanted adult MSCs were shown to exert a remarkably beneficial effect in different injury models, regardless of their poor *in vivo* engraftment and long-term differentiation. This therapeutic effect, mediated by MSC paracrine activity (Prockop and Oh, 2012; Kean et al., 2013), was exploited in a variety of preclinical models, among the others the myocardial infarction, the hind-limb ischemia, and the acute renal failure (Heederik et al., 1992; Gnecchi et al., 2005; Tögel et al., 2005). The relevance of the MSC paracrine potential inspired the definition of these cells as “site-regulated, multidrug dispensaries, or injury drugstores” (Caplan and Correa, 2011).

It was also demonstrated that the intrinsic capacity of MSCs to activate endogenous regenerative mechanisms and to induce the mobilization of host cells was critically dependent on their commitment level, highlighting the importance of carefully investigating differences in the soluble morphogens used as culture medium supplements. The addition of basic-fibroblast growth factor (bFGF or FGF-2) to primary bone marrow-derived MSC cultures was a key element to select *in vitro* specific MSC subpopulations with potential to induce a host regenerative response *in vivo* (Tasso et al., 2012).

Within the specific components present in the stem cell-conditioned medium, much interest has gained the extracellular vesicles released by cells (EV, including microvesicles and exosomes) (Camussi et al., 2013; Quesenberry et al., 2014). EV can transfer, in addition to proteins and lipids, also genetic information such as mRNA and microRNA (miRNA) (Phinney et al., 2015; Zhang et al., 2016). In particular, miRNAs are small non-coding RNA exerting on target cells a post-transcriptional regulation of proliferation, differentiation, and death. Indeed, it was suggested that stem cells promote healing and tissue regeneration at the injury site also by delivering specific transcripts to target cells *via* EV. Extracellular vesicles have the advantage of (i) being immunologically inert, (ii) having the capacity to carry a significant amount of bioactive molecules, (iii) protecting them from enzymatic degradation because of the lipid bilayer and, at the same time, (iv) being able to transfer them to other cells by bypassing the hydrophobic membrane barrier. Therefore, EV have all properties for being an almost ideal vehicle for paracrine therapy.

Recent studies indicated that the cross talk between MSCs and cells of the innate immunity could be carried out by secreted EV. In particular, EV isolated from the conditioned medium of MSC harvested under both normoxic and hypoxic culture conditions acted as mediators of the dynamic interplay between MSCs and cells of the innate immunity both *in vitro* and *in vivo* in a mouse model of skeletal muscle regeneration (Lo Sicco et al., 2017). EV effectively triggered the macrophage proliferation and polarization from an M1 to an M2 phenotype. More recently, we provided the first characterization of hAFS-secreted extracellular vesicles as paracrine mediators endowed with regenerative miRNAs and supporting pro-survival, proliferative, and anti-inflammatory effects on target cells (Balbi et al., 2017). The hypoxic preconditioning induced an intensified release of EV enriched with miRNAs involved in different stages of the healing process. Direct administration of EV in a cardiotoxin-induced skeletal muscle injury reduced the inflammatory response, upregulated key markers of alternative activation patterns, and accelerated the expression of myogenic markers.

Interestingly, exosomes released in the extracellular compartment were identified as one of the effectors also of platelet activity (Torreggiani et al., 2014).

## Unlocking the Hidden Potential of Endogenous Progenitors

From studying limb regeneration in amphibians and observing the few regenerative models in mammals, including humans, we learned that the process leading to complete tissue regeneration can occur through paracrine signals recruiting to the wound site: (i) bone marrow-derived and tissue-specific stem/progenitor cells and (ii) de-differentiated cells deriving from lineage committed cells.

### Activation and Mobilization of Tissue Resident Progenitor Cells

Pericytes are perivascular multipotent cells found in close relation to the walls of small blood vessels within connective tissues (Bergers and Song, 2005). They are a heterogeneous population with specific differences depending on their embryonic origin (neuroectodermal or mesodermal) and the type of vessels (venules or capillaries) they reside in. Markers used to identify pericytes, such as alpha smooth muscle (αSMA), PDGF receptor (PDGFRβ), chondroitin sulfate proteoglycan 4, CD146, NG2, and others, are differently distributed among the pericyte population also related to their anatomical location (Armulik et al., 2011). Most of these markers are not exclusively expressed by pericytes, thus their specific isolation depends on the co-expression of different markers. Several studies have shown that, in addition to stabilize vessels, pericytes perform other functions such as controlling vessel development, maturation, and permeability, regulating blood flow and coagulation, maintaining the blood–brain barrier, and regulating phagocytosis and lymphocyte activation (Goodkin and Benning, 1990; Fabry et al., 1993; Geevarghese and Herman, 2014), as well as playing a major role in regeneration and repair of several tissues in response to injury (Birbrair et al., 2015). All vascularized tissues contain pericytes exhibiting progenitor properties, but the *in vivo* potential and developmental interactions between pericytes, mesenchymal stem/stromal cells, and tissue-specific progenitors, remain mostly unknown. Notably, pericytes have been considered similar to MSCs since they express some of the MSC markers both *in vitro* and *in vivo*. As blood vessels are present in almost all organs, it was also suggested that pericytes correspond to tissue-specific adult stem cells (Sacchetti et al., 2007; Caplan, 2008; Corselli et al., 2010). Even though pericytes are key players and regulators of vascular morphogenesis and their anatomical apposition and their sharing the same basement membrane ease interactions between pericytes and endothelal cells, the pericyte role is not fully understood. A collection of articles on nature and functions of pericytes and their *in situ* activation for tissue regeneration/repair was very recently published (Madeddu and Peault, 2017). Pericyte regenerative potential has been exploited for therapy of several pathologies, including cancer. Given their adaptability for applications of tissue engineering, pericytes were also proposed for the repair of several tissues and organs, including vascular grafts, skeletal muscles, heart, skin, bone, and cartilage. Moreover, pericytes were applied in diverse orthopedic models, both ectopic and orthotopic. Pericyte implantation resulted in improved tissue healing by both direct and indirect mechanisms. Several studies are presently being performed on factors released by pericytes, including cytokines, microRNA, and microvesicles, that are possibly involved in the cross talk between pericytes and other cell types.

However, in contrast to the many positive effects induced by the pericyte activation, in some cases, the extreme adaptability of these cells could be a brake to tissue regeneration and repair. A recent study by Dulauroy et al. (2012) identified a pericyte derived myofibroblast progenitor by the expression of the cell membrane-associated protease, ADAM12, and revealed a significant role for this progenitor in the tissue fibrosis observed after skin and skeletal muscle injury. The genetic ablation of ADAM12 inhibited fibrosis, prevented the formation of a scar acting as a barrier precluding interactions of epithelial and underlying cells thus impairing the neo-tissue formation, and allowed tissue repair.

### Recruitment of Circulating Stem/Progenitor Cells with Healing Potential

The idea of adult stem cells behaving as cell progenitors restricted to their tissue of origin and being the only ones involved in tissue regeneration must be reconsidered. Several publications report that, in response to a tissue damage and under the influence of various pathological stimuli, tissue-specific or bone marrow-derived stem/progenitor cells are mobilized to the PB and migrate to the wound site, where they exert a master role during wound healing, being directly involved or by enhancing the activation of resident cells. All stem/progenitor cells from the PB described so far, were specifically isolated in pathological conditions or following exogenous stimulation such as using mobilizing factors (Otsuru et al., 2008; Ciraci et al., 2011). Hematopoietic stem cells are the archetype of resident stem cells that can be mobilized and used for transplantation therapies (Copelan, 2006). EPCs circulating in the PB are effectively involved in angiogenesis and vasculogenesis processes (Basile and Yoder, 2014) although they present restricted differentiation capacity.

The presence of very small-sized stem cells with pluripotent characteristics in the bone marrow and other adult organs was reported (Kucia et al., 2006). However, the real existence of such cell population is very controversial, possibly due to debatable flow cytometry analysis and the adopted cell isolation procedures (Miyanishi et al., 2013), and there was no evidence for the presence of stem/progenitor cells of mesenchymal nature circulating in the PB in physiological conditions. Recently, we reported the identification of a rare cell population of circulating cells derived from the PB of healthy mice and actively participating in the healing process (CH cells) (Lo Sicco et al., 2015). Indeed, CH cells behaved as connective tissue precursors able to integrate in wounded tissues and to appropriately differentiate into a broad spectrum of tissue-specific cells. These progenitor cells were identified as small cells characterized by the pan-hematopoietic CD45 antigen lack of expression, as well as of the markers expressed by differentiated blood cells, and of markers typically associated with well-defined progenitors circulating upon injury. The analysis of the global transcriptional profile of the purified CH cells revealed the expression of markers expressed also by epiblasts during the embryogenesis process. As a whole, our findings support the concept of CH cells as key effectors of the body innate regenerative potential and suggest new ways of approaching tissue regeneration. In light of this study, one can also consider the intriguing possibility that a specific recruitment of endogenous progenitors with mesenchymal characteristics could guaranty the physiological cell turnover in connective tissues throughout the lifespan of the individual and, at the same time, enhance tissue repair or regeneration when a tissue injury occurs. However, this hypothesis could open a completely new field of research and additional extensive investigations are required to confirm the hypothesis.

### Reprogramming of Tissue Differentiated Cells

As stated before, amphibians are good models to investigate mechanisms of tissue and organ regeneration. We already mentioned how, for limb regeneration, the newt switches from a juvenile stem/progenitor-based process to an adult dedifferentiation-based mode where cells from the different limb tissues strictly regenerate the same type of tissues (Tanaka et al., 2016). Another well-known example of organ regeneration, through reprogramming of differentiated cell, is the zebrafish heart. This organ can fully regenerate in few weeks following severe injury by the activation of resident cardiac progenitor cells influencing the dedifferentiation, proliferation, and maturation of the surviving cardiomyocytes (Lepilina et al., 2006). However, although cellular and molecular mechanisms of regeneration are currently being investigated also in a variety of other contexts (Tanaka and Reddien, 2011), our understanding of epigenetic reprogramming occurring during regeneration is only at the beginning (Katsuyama and Paro, 2011). Indeed, a heated debate is still going on about the hypothesis that, in mammals including humans, after a tissue injury, differentiated cells can revert to a pre-differentiation stage, proliferate, and contribute to the restoration of the tissue of origin, in addition to the canonical view that tissue resident or recruited stem/progenitor cells are responsible of regenerative effects. However, although sometimes the two mechanisms have been presented as mutually exclusive, actually they can co-exist and can act synergistically. Indeed, there is a rising evidence that both mechanisms (i) local induction of proliferation of progenitor/stem cells (Crisan et al., 2011; Chen et al., 2015; Sacchetti et al., 2016) and (ii) dedifferentiation, proliferation, and reprogramming of mature differentiated or committed cells (Jopling et al., 2011; Riddell et al., 2014; Szibor et al., 2014; Tarlow et al., 2014; Wahlestedt and Bryder, 2014) play a role in the repair/regeneration of damaged tissues also in mammals, contributing with a major or minor role, depending on the individual age. In particular, in humans, recent evidence from studies on phenotype conversion and proliferation of cardiomyocytes in postnatal life (Zacchigna and Giacca, 2014; Kimura et al., 2015) as well as adult endochondral (von der Mark and Zhou, 2016) and articular (Jiang et al., 2016) chondrocytes indicates that replication of already differentiated cells might play a critical role during tissue and organ healing, on top of recruitment and activation of stem/progenitor cells.

In our group, we investigated the effect of the factors present in the PL on reprogramming differentiated cells either with or without concomitant presence of inflammatory cytokines, using cultures of confluent terminally differentiated human cells, in a stage of basic metabolism as a model. PL induced an initial pro-inflammatory response along with an increased migratory capacity on a keratinocyte cell line (El Backly et al., 2011). Moreover, following PL stimulation, chondrocyte otherwise not responding to standard culture conditions (fetal calf serum supplement) responded with a strong induction of proliferation while maintaining their chondrogenic potential (Pereira et al., 2013). In quiescent osteoblasts, PL triggered an initial pro-inflammatory activity along with IL-6, IL-8 secretion, and simultaneous induction of COX-2 and PGES, eventually leading to PGE_2_ synthesis and resolution of inflammation (Ruggiu et al., 2013). The inflammatory phase was paralleled by cell morphology changes and a cell cycle re-entry leading to cell proliferation. PL stimulated cells reached confluence at a higher cell density still retaining their differentiation capability.

### Pharmacological Induction of Tissue Regeneration

The long-term goal of regenerative medicine is to adopt cell-inspired strategies to identify ideal drug formulation in order to mimic the physiological inducers of tissue healing and regeneration so to mobilize the different cell players mastering these processes. This will allow a large-scale production of “off the shelf” therapeutic agents for tissue repair and regeneration, with major benefits for a large number of patients. Research in this area is still in its infancy and very few sporadic reports on drug-induced activation of genes and pathways involved in tissue regeneration exists (Zhang et al., 2015; Zhao et al., 2015; Fan et al., 2016). However, these potentially high potent agents being associated with a series of possible and still partly unknown risks, major investments in basic science, preclinical, and clinical studies are needed before this ambitious goal could be reached.

## Conclusion

Despite the big interest of the scientific and medical community in the possibility of restoring the body endogenous regenerative potential following an injury and the increasing number of reports on this topic, further studies are needed in order to pinpoint specific mechanisms and discrete masters regulators needed to obtain true tissue regeneration, on top of basic repair. Eventually, by identifying the relevant signaling pathways and the key molecules involved, we will be able to envisage an *advanced medicinal product* to be exploited for future regenerative medicine.

## Author Contributions

RC: conceived the review, wrote part of the review, and reviewed and edited the submitted text. SB, FD, MM, and RT: wrote part of the review and reviewed and edited the submitted text.

## Conflict of Interest Statement

The research was conducted in the absence of a conflict of interest. However, RC and MM declare that they are shareholders of Biorigen Srl, a spin off company of the University of Genova, with an interest in regenerative medicine.
